# Discovery of Genomic Regions and Candidate Genes Controlling Root Development Using a Recombinant Inbred Line Population in Rapeseed (*Brassica napus* L.)

**DOI:** 10.3390/ijms23094781

**Published:** 2022-04-26

**Authors:** Lieqiong Kuang, Nazir Ahmad, Bin Su, Lintao Huang, Keqi Li, Hanzhong Wang, Xinfa Wang, Xiaoling Dun

**Affiliations:** 1 Oil Crops Research Institute of the Chinese Academy of Agricultural Sciences/Key Laboratory of Biology and Genetic Improvement of Oil Crops, Ministry of Agriculture and Rural Affairs, Wuhan 430062, China; kuanglieqiong@163.com (L.K.); nazir_aup@yahoo.com (N.A.); su13297095753@163.com (B.S.); hlt1406859197@outlook.com (L.H.); likeqi1218@sina.com (K.L.); wanghz@oilcrops.cn (H.W.); 2 Hubei Hongshan Laboratory, Wuhan 430070, China; 3 College of Agriculture, Yangtze University, Jingzhou 434023, China

**Keywords:** rapeseed, root development, linkage analysis, major QTL, fine mapping

## Abstract

Marker-assisted selection enables breeders to quickly select excellent root architectural variations, which play an essential role in plant productivity. Here, ten root-related and shoot biomass traits of a new F_6_ recombinant inbred line (RIL) population were investigated under hydroponics and resulted in high heritabilities from 0.61 to 0.83. A high-density linkage map of the RIL population was constructed using a *Brassica napus* 50k Illumina single nucleotide polymorphism (SNP) array. A total of 86 quantitative trait loci (QTLs) explaining 4.16–14.1% of the phenotypic variances were detected and integrated into eight stable QTL clusters, which were repeatedly detected in different experiments. The codominant markers were developed to be tightly linked with three major QTL clusters, *qcA09-2*, *qcC08-2*, and *qcC08-3*, which controlled both root-related and shoot biomass traits and had phenotypic contributions greater than 10%. Among these, *qcA09-2*, renamed *RT.A09*, was further fine-mapped to a 129-kb interval with 19 annotated genes in the *B. napus* reference genome. By integrating the results of real-time PCR and comparative sequencing, five genes with expression differences and/or amino acid differences were identified as important candidate genes for *RT.A09*. Our findings laid the foundation for revealing the molecular mechanism of root development and developed valuable markers for root genetic improvement in rapeseed.

## 1. Introduction

Plants rely on their root system architecture (RSA), which usually denotes the three-dimensional spatial distribution of the root system in the growth substrate, to absorb water and mineral nutrients [1,2]. Previous studies have revealed that the improvement of the RSA, such as the root volume, number of roots, root surface area, and root length, increased resource bioavailability and thus boosted the biomass and yields of crops [3,4]. However, most breeding efforts to boost crop yields were based on aboveground traits, and the roots are still underutilized in crop improvement as the ‘hidden half’ of the plant [5]. In-depth insight into the genetic mechanism of the RSA is required to accelerate the breeding of crops with high biomass and yields.

Quantitative trait locus (QTL) analysis based on genome-wide molecular linkage analysis is an important method for detecting and interpreting the genetic factors that affect quantitative traits [6]. As reported, hundreds of QTLs affecting RSA in controlled environments or in the field have been identified. Examples include QTLs controlling the root length, root angle, root depth, and lateral root growth in rice [7,8,9]; the root architecture and adventitious root development in soybeans [10,11]; and seedling root traits and crown root traits in maize [12,13]. Previous reports also found that several identified loci controlling RSA were colocalized with QTLs for nutrient uptake [7,10,14], water uptake [15,16], drought stress [17,18], and salt tolerance [19,20]. Hence, the improvement of root traits can lead to higher resistance to abiotic stresses, higher water and nutrient use efficiency, and increased crop yields. Furthermore, some of the identified loci have been shown to be available for the genetic improvement of RSA. For example, *DEEPER ROOTING 1* (*DRO1*) was reported to control both the root depth and root growth angle in rice [9]. The loss of function of *qSOR1* (*quantitative trait locus for SOIL SURFACE ROOTING 1*), a *DRO1* homologue, enhances rice yields in saline paddy soils by constructing a shallower root growth angle [3]. However, the data available on the genetic regulation of root traits in the field and their relationships with yields are limited because root research in the field is complicated by the soil profile, structure, and composition [21,22]. High-performance phenotyping remains a bottleneck for the genetic analysis of root traits. Hydroponics, an effective approach for phenotyping root traits to address this bottleneck, allows for quick and low-cost screening of root traits in a large panel of plants [23,24].

Rapeseed, a globally cultivated crop, is not only the world’s third-largest source of edible vegetable oils but also the world’s second-largest source of biodiesel [25,26]. Vegetable oils are the main raw materials for biodiesel production, accounting for more than 70% of the global demand [27]. In addition, rapestraw is very suitable for producing liquid biofuel, particularly ethanol, because of its high lignocellulose component [28]. Increasing rapeseed yields and biomass through genetic improvement will create high economic value accompanying the growing demand for edible oil and fuel in the global market. Recently, there have been many reports regarding genetic studies of the RSA in *B. napus* L., including the significant loci for root vigor, primary root length, lateral root length, number of lateral roots, and root weight [29,30,31], as well as the genetic dissection of root traits related to nutrient efficiency [32,33,34]. However, genetic improvement and map-based gene cloning for root development have been limited to only a few loci in rapeseed. This study revealed available major QTLs for root-related and shoot biomass traits in *B. napus* using QTL mapping in a newly constructed recombinant inbred line (RIL) population. Segregating populations derived from the residual heterozygous lines (RHLs) in the RIL population were used to fine map the major QTL *RT.A09*. Our study provided candidate genes of *RT.A09* via the map-based gene cloning method and developed codominant molecular markers for marker-assisted selection (MAS) breeding of root traits.

## 2. Results

### 2.1. Phenotypic Performances of the RIL Population and Its Parents

**Figure 1:**
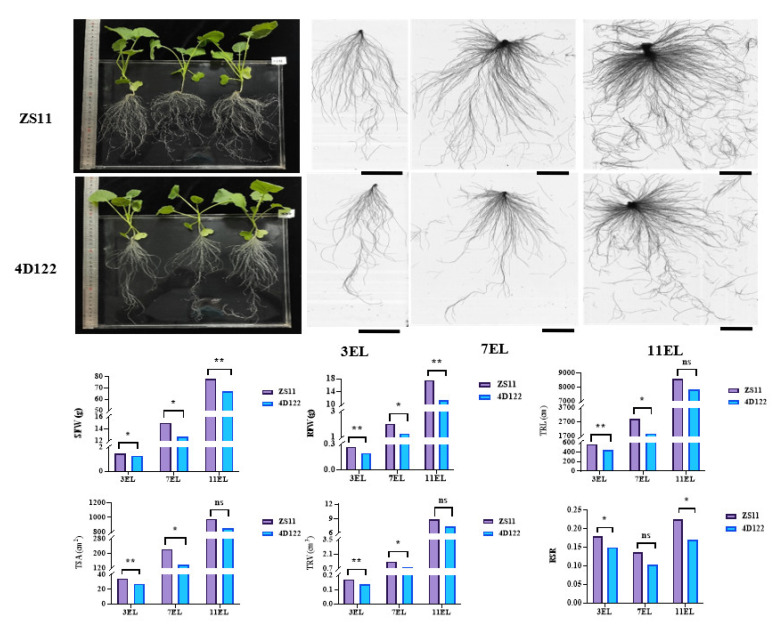
Comparison of phenotypic characteristics of two parents, ZS11 and 4D122. The whole plant performance of the two parents was cultured on the 18th day of the three expanding leaves (3 EL). The root scanning images of the two parents were present at 18, 32, and 46 days old with three, seven, and eleven expanding leaves (3EL, 7EL, and 11EL), respectively. The bars are 5 cm. ** and * indicate significance at the 1% and 5% levels of probability, respectively, while ns indicates non-significant difference.

An RIL population containing 236 F_6_ lines was developed from a cross between the parents *B. napus L.* ZhongShuang11 (ZS11) and 4D122. The root-related and shoot biomass traits of the two parents, including the shoot fresh weight (SFW), root fresh weight (RFW), root-shoot ratio (RSR calculated by RFW/SFW), and five root morphological traits (primary root length (PRL), total root length (TRL), total root surface (TSA), total root volume (TRV), and total number of roots (TNR)), were evaluated under hydroponics at three vegetative stages with three expanding leaves (3 EL), 7 EL, and 11 EL, respectively. The parent ZS11 showed significantly larger SFW, RFW, TRL, TSA, TRV, and RSR than the other parent 4D122 at no less than two examined stages, particularly at the 18 days with three expanding leaves (3 EL stage) (Figure 1). These results suggested that the two parents contributed different genetic effects towards the examined traits.

**Figure 2:**
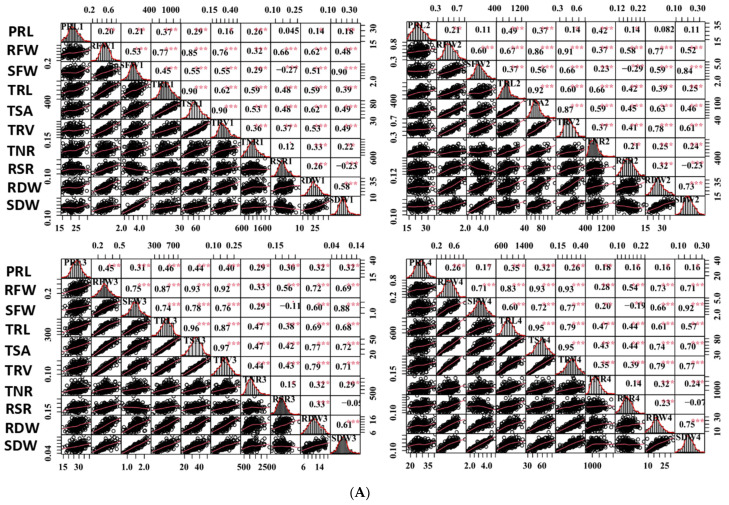

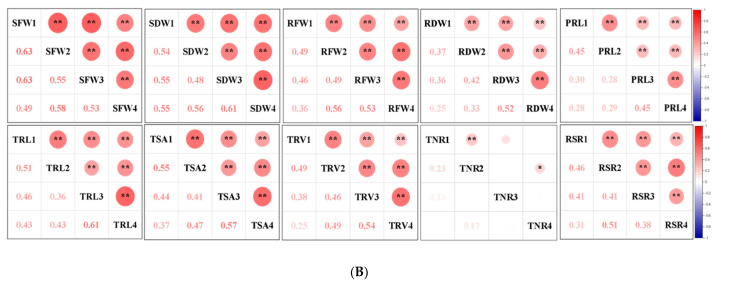
Correlation analysis of captured traits. (**A**) Correlations of captured traits in each experiment among RILs. For each trait, the frequency distributions of the adjusted means (diagonal), scatterplots (below diagonal), and values of the correlation coefficients (above diagonal) between pairs of traits are shown. (**B**) Correlations of each captured trait among the four experiments. Red and blue indicate positive and negative correlations, respectively. ***, ** and * denote significance at the 0.1%, 1% and 5% levels of probability among the RILs, respectively.

Then, phenotypic characteristics of the ZS11/4D122 RIL population, including the SFW, RFW, RSR, PRL, TRL, TSA, TRV, TNR, shoot dry weight (SDW), and root dry weight (RDW), were evaluated in four independent experiments. In almost all of the four experiments, the two parents differed significantly in several root traits, including the SFW, RFW, TRL, TSA, TRV, and RSR, except for SFW, TSA, and TRV in the first experiment and TRL in the second experiment (Table 1). The statistical data, frequency distribution, and Pearson correlation coefficients of these traits in the RIL population are displayed in Table 1 and Figure 2. Broad phenotypic variations of all examined traits were noticed within the RIL population, as indicated by the coefficient of variation values ranging from 15.5% to 35.7% and the considerable differences between the maximums and the minimums (Table 1). The population skewness and kurtosis for the vast majority of the examined traits were smaller than one in the four replications, indicating that these traits were segregated according to a skew-normal or normal distribution model (Table 1 and Figure 2A). The captured traits resulted in high broad-sense heritabilities (h^2^) ranging from 0.61 to 0.83. Notably, the SFW and RSR gave higher h^2^ values of 0.83 and 0.80, respectively. Regarding root traits, the RFW, TRL, TSA, and TRV had approximate h^2^ greater than those of the TNR and PRL (Table 1).

Most of the studied traits exhibited a strong positive and significant correlation with other traits in each experiment among the RILs, except for the RSR and PRL. The RSR showed negative correlations with the SFW and SDW, and the PRL displayed no or low correlations with other traits (Figure 2A). The RFW revealed highly significant correlations with the SFW (0.53–0.75), RSR (0.62–0.77), and several root morphological traits, including the TRL (0.67–0.87), TSA (0.85–0.93), and TRV (r = 0.76–0.93); however, it had relatively low correlations with the TNR (0.28–0.37) and PRL (0.20–0.45). Similarly, the SFW was found to be highly significantly correlated with the TRL (0.37–0.74), TSA (0.55–0.78), and TRV (0.55–0.77). These findings supported the feasibility of the synergistic improvement of the root system and biomass in rapeseed. Furthermore, all the investigated traits showed a high correlation (0.25–0.63) among the four experiments, except for the TNR (0.03–0.23) (Figure 2B). The high h^2^ and correlations among the four experiments demonstrated the genetic stability of the traits and produced high-quality statistical data to perform QTL analysis.

### 2.2. The Construction of the High-Density Genetic Linkage Map

**Figure 3:**
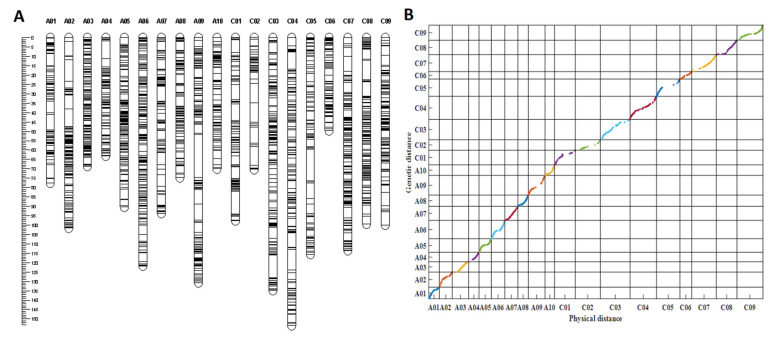
Information of the genetic linkage map. (**A**) The genetic linkage map with 19 linkage groups constructed by 2089 bins. (**B**) Collin-earity analysis of the genetic distances and physical distances of the markers in the linkage map; different colors represent different chromosomes or linkage groups.

The RIL population and the two parents were first genotyped by a *B. napus* 50 K Illumina Infinium SNP array, which were developed by Greenfafa Biotech Co., Ltd. (Wuhan, China), with 28,491 SNPs mapped on the *B. napus* *Darmor-bzh* genome [35]. Of these, 10,721 SNPs detected polymorphisms between the parental lines ZS11 and 4D122. After filtering, 8703 SNPs that produced 2247 bins (the markers with the same genetic positions were integrated into a “bin”) were subjected to JoinMap 4.0 software for linkage analysis. As a result, 2089 bins covering the 19 linkage groups resulted in a total genetic distance of 1826.03 cm and an average genetic distance of 0.87 cm (Table S1, Figure 3A). The average genetic distance varied from 0.51 cm (A03) to 1.43 cm (C02). The maximum genetic gap (22.86 cm) between the markers was found on A09, while the minimum (3.43 cm) was observed on the C06 linkage group. The C subgenome had more SNP markers but with smaller bins and a higher average marker distance than the A subgenome, indicating that the C subgenome recombination frequency was lower due to smaller bins than that of the A subgenome in the RIL population (Table S1).

Collinearity analysis was performed to evaluate the quality of the genetic linkage map based on the genetic distances and physical distances of the markers in the linkage map and the *B. napus* genome, respectively. In total, 1943 out of 2089 bins were successfully mapped to the *B. napus* reference *Darmor-bzh* genome [35]. According to (Figure 3B), drawn by MATLAB software, almost all markers in each linkage group were collinear with their physical positions on the *B. napus* reference genome, with an average correlation coefficient of 0.962 and the correlation coefficient ranging from 0.915 (A10) to 0.997 (A03) (*p* < 0.0001, Table S2), indicating the high quality of the linkage map.

### 2.3. Identification of the Major QTLs for Root and Shoot Traits

Through composite interval mapping (CIM) analysis, 86 QTLs for the evaluated traits were detected within the four experiments, contributing 4.2–14.1% of the observed phenotypic variance (R2) to the examined traits (Table S3). Among these QTLs, 52 QTLs had a negative additive effect and carried the enhancing alleles from the parent ZS11. In contrast, 34 QTLs with a positive additive effect and favorable alleles from 4D122 were reported. Concerning the root-related QTLs, 54 loci—8 for the RFW, 7 for the TRL, 9 for the TSA, 9 for the TRV, 3 for the TRN, 13 for the PRL, and 5 for the RDW—were identified. For the shoot biomass, a total of 26 loci were observed: 13 for the SFW and 13 for the SDW. For the RSR, six loci were observed.

QTL clusters are genomic regions to which no less than two QTLs related to different traits are colocalized, commonly referred to as QTL hot spots [36]. Most pairs of root and shoot variables had strong relationships in this study, implying that QTLs with pleiotropy for these traits might be identified. By QTL meta-analysis, 73 identified loci (85%) were integrated into 15 unique pleiotropic QTL clusters based on the overlapping CIs for different traits or at different repetitions (Table S4). All of these QTL clusters displayed the same sources of additive effects. According to the traits contained in these loci, the QTL clusters could be classified further into three types: root-specific QTL clusters (including 7 QTL clusters: qcA01-1, qcA01-2, qcA10-1, qcC02-2, qcC02-3, qcC06-1, and qcC08-1), shoot-specific QTL clusters (including 3 QTL clusters: qcA09-1, qcC02-1, and qcC03-1), and root and shoot common QTL clusters (including 5 QTL clusters qcA09-2, qcC01-1, qcC04-1, qcC08-2, and qcC08-3) (Table S4). Notably, 8 out of the 15 QTL clusters, including the QTLs for one trait that could be detected in at least two repetitions, were considered stable QTL clusters (Table 2). The genomic regions of these stable clusters corresponded to their physical positions in the genome sequence of Darmor in Table 2.

**Figure 4:**
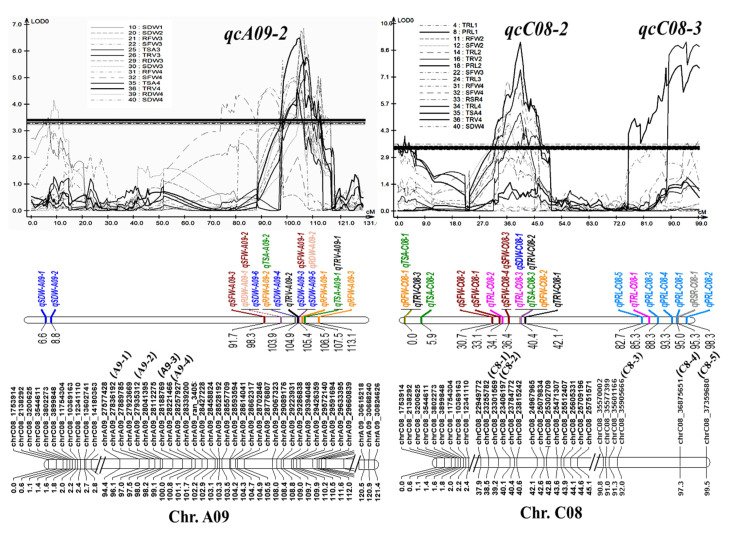
Information of the three major QTL clusters, *qcA09-2*, *qcC08-2*, and *qcC08-3*. Three major QTL clusters with their involved QTLs, LOD curves, and SNP markers at the corresponding linkages. SNPs noted with other names, A9-1, A9-2, A9-3, A9-4, C8-1, C8-2, C8-3, C8-4, and C8-5, were converted into PARMS markers.

Three stable QTL clusters, qcA09-2, qcC08-2, and qcC08-3, composed of 15, 10, and 7 QTLs (Table S4), with phenotypic contributions of 10.8%, 14.1%, and 13.9% (greater than 10%), respectively, were considered as major QTL clusters that could be used for further marker-assisted selection (MAS). All three were root and shoot common QTL clusters, suggesting their roles in the synergistic improvement of roots and biomass. To facilitate the utilization of the three QTL clusters, several SNP markers, including A9-1, A9-2, A9-3, and A9-4 for qcA09-2, C8-1 and C8-2 for qcC08-2, and C8-3, C8-4, and C8-5 for qcC08-3, were developed using the penta-primer amplification refractory mutation system (PARMS) method (Table S5) and were verified to be tightly linked with the corresponding QTLs by genotyping the two parents and the RIL population (Figure 4). The codominated PARMS markers could be used for MAS with an excellent root system in the future.

### 2.4. Fine Mapping of the Major QTL RT.A09

**Figure 5:**
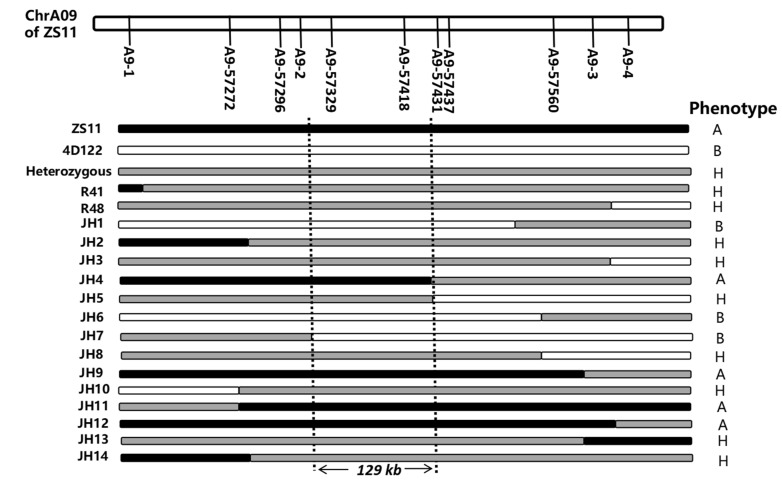
Fine mapping of the QTL *RT.A09*. The genotype and phenotype of recombinants using 11 developed PARMS markers of *RT.A09*. “A” and “B” represent that the offspring phenotypes of the recombinant line were similar to NIL-A and NIL-B, respectively, of its sister lines. “H” represents that the offspring phenotypes of the recombinant line were separated according to their genotypes.

Furthermore, the major QTL cluster *qcA09-2*, located at 25.77–28.22 Mb on A09, was renamed *RT.A09*. A map-based gene cloning approach with a large separate population and more molecular markers in the initial positioning interval was performed to further map the target gene. Based on the similar genetic background, the F_6_ sister lines of residual heterozygous lines (RHLs) could be used as near-isogenic lines (NILs) to map *RT.A09*. Eight F_6_ RHLs in the initially located interval of *RT.A09* were selected from the RIL population (Table S6). The four PARMS markers (A9-1, A9-2, A9-3, and A9-4) linked to *RT.A09* were used for genotyping the 71 sister lines. The root and shoot traits of these sister lines were evaluated at the 3 EL stage by progeny tests. For the six RHLs (R4, R19, R26, R78, R159, and R183) with the separation of NIL-A (the genotype from ZS11) and NIL-B (the genotype from 4D122) from A9-1 to A9-5, NIL-A displayed significantly larger values than NIL-B in the RFW, SFW, TRL, TSA, and TRV (Figure S1). The results further verified the genetic effects of *RT.A09* for root and shoot traits. Moreover, according to the genotypes and phenotypes of two RHLs, R41 and R48, which were partially heterozygous from A9-1 to A9-4 and displayed phenotypic differences among their NILs, *RT.A09* was flanked by markers A9-1 (chrA09_27836192) and A9-3 (chrA09_28188769), consistent with A09: 57.18–57.59 Mb in the ZS11 genome (http://cbi.hzau.edu.cn/bnapus/, accessed on 18 October 2021) (Figure S2, Figure 5).

To precisely map *RT.A09*, seven new PARMS markers using insertions/deletions or SNPs between parents were developed (Table S5). The flanking SNP markers A9-1 and A9-3 and the seven new markers were used to screen the NIL population with 1137 offspring obtained from the selfing of RHLs. Based on the phenotype evaluation of 14 recombinations that were verified by progeny testing, *RT.A09* was subsequently localized to an interval of 129 kb between markers A9-2 and A9-57431 and cosegregated with A9-57329 and A9-57418 (Figure 5).

### 2.5. Candidate Gene Screening of the QTL-RT.A09 Interval

Nineteen genes, from BnaA09G0558900ZS to BnaA09G0560700ZS, were annotated from the fine-mapping region of *RT.A09* according to the ZS11 genome database (http://cbi.hzau.edu.cn/bnapus/, accessed on 18 October 2021) (Table 3). First, the expression profiles of the 19 genes were examined using the *B. napus* transcriptome information resource database (BnTIR, http://yanglab.hzau.edu.cn/BnTIR, accessed on 18 October 2021)) with 90 *B. napus* organs. Eight genes (BnaA09G0558900ZS, BnaA09G0559000ZS, BnaA09G0559400ZS, BnaA09G0559700ZS, BnaA09G0560200ZS, BnaA09G0560500ZS, BnaA09G0560600ZS, and BnaA09G0560700ZS) with no or negligible expression in the roots, vegetative rosette organs, stems, and leaves were excluded from the candidate genes based on their expression patterns (Table S7).

**Figure 6:**
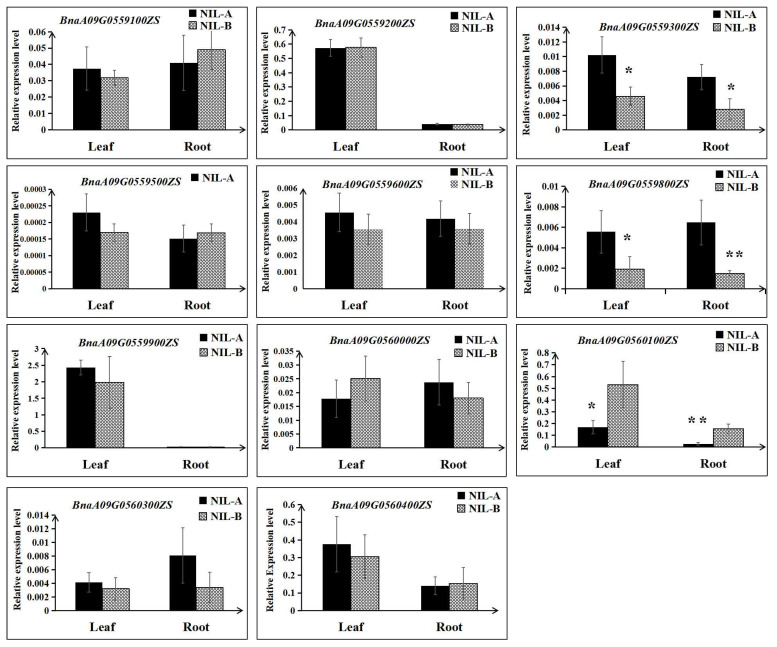
Relative expression levels of the eleven candidate genes between NIL-A and NIL-B. The mean and the standard deviation were calculated using three biological replicates. ** and * indicate significant differences with *p* < 0.01 and *p* < 0.05, respectively.

Then, the expression differences of the remaining 11 genes were detected using RNA extracted from roots and leaves at the 3 EL stage of NIL-A and NIL-B from R41 by qRT–PCR. As shown in Figure 6, for both roots and leaves, the expression levels of BnaA09G0559300ZS and BnaA09G0559800ZS in NIL-A were higher than those in NIL-B; in contrast, the expression level of BnaA09G0560100ZS in NIL-A was lower than that in NIL-B (Figure 6). Furthermore, comparative sequencing of the 11 genes was performed to detect the coding sequence differences between ZS11 and 4D122. As a result, no sequence variations for four genes (BnaA09G0559900ZS, BnaA09G0560000ZS, BnaA09G0560100ZS, and BnaA09G0560300ZS) were detected. Moreover, of the remaining seven genes with SNPs or insertions/deletions, only three genes (BnaA09G0559200ZS, BnaA09G0559300ZS, and BnaA09G0559600ZS) displayed differences in amino acids, and BnaA09G0559600ZS even showed a frameshift mutation (Table 3, Figure S2). To integrate the results of qRT–PCR and comparative sequencing, the five genes BnaA09G0559200ZS, BnaA09G0559300ZS, BnaA09G0559600ZS, BnaA09G0559800ZS, and BnaA09G0560100ZS could be considered candidate genes of *QTL-RT.A09* until further validation is performed.

## 3. Discussion

Accurate and efficient root phenotyping is the major factor limiting the genetic dissection and improvement of RSA due to the heterogeneity of nutrients and water availability and distribution status, soil density, salinity and temperature, and microorganisms interactions, which amplify environmental noise. Indoor growing methods using artificial media (e.g., hydroponics) have become a popular means to help researchers conduct more accessible imaging and collect samples more easily [8,9,37]. This study used a modified hydroponics system suited for assessing a large scale of genotypes at different vegetative stages [24]. Various traits revealed high h^2^ ranging from 0.61–0.83, suggesting that the observed traits were under less environmental influence. Similar results with high h^2^ (0.63–0.87) were also noted for root-related traits in our previous study with three environments [24]. The high heritability ensures the trait’s repeatability, which is the fundamental criterion for plant breeders during selection [38]. In accordance with previous reports [23,30,32], strong and positive correlations among different root-related traits indicated that these traits were not expressed independently of one another.

Genetic linkage mapping has become a useful tool for genetic improvements of complex traits and functional gene cloning in crops [39,40]. A high-density linkage map is helpful to improving QTL localization and effect precision, particularly for tiny and medium-sized QTLs [41,42]. Our study created a high-density genetic map with an average distance between less than 1.0 cm loci. Furthermore, the high level of collinearity with the *B. napus* reference genome indicated that the map was of excellent quality and accuracy. Hence, QTL analysis was performed to identify significant loci in the newly constructed rapeseed RIL population. Based on high correlations between the examined traits, researchers were able to identify pleiotropic or closely linked genes by using QTL clusters [43]. A total of 86 QTLs (15 QTL clusters), with eight stable QTL clusters, were identified in this study. Colocalization of these QTLs implied the benefit of this genomic region in breeding efficiency for numerous elite traits [44]. In this study, three major QTL clusters, *qcA09-2, qcC08-2*, and *qcC08-3*, with phenotypic contributions greater than 10%, were found to be of value for further study of gene cloning and MAS. Furthermore, *qcA09-2*, located on 25.77–28.22 Mb on A09 of the *Darmor-bzh* reference genome, has also been found to be related to root and shoot traits in a natural population by a genome-wide association study and additionally has been found to be associated with water use efficiency and drought tolerance in rapeseed [45,46], indicating the prevalence of this variation in rapeseed resources. 

RHLs have been proven to be an effective tool for validating and fine-mapping QTLs [46]. In soybean, two maturity loci (E2 and E3) using an RHL-derived map-based cloning strategy were reported [46]. We also mapped the QTL cluster *qcA09-2*, renamed *RT.A09*, into a 129-kb interval using RHLs derived from the RIL population. The cosegregated markers A9-57329 and A9-57418 of *RT.A09* can be employed to improve RSA using MAS. *B. napus* genome sequencing and expression databases would aid in the development of molecular markers and the understanding of gene function, regulation, and expression [47,48]. Nineteen putative genes were annotated in the located region of *RT.A09* based on the reference genome. Analysis of the expression patterns and comparison of the sequencing showed five candidate genes with different expression levels between NILs and/or with CDS differences between parents. *BnaA09G0559300ZS* and *BnaA09G0560100ZS*, which express differences between NILs, encode *auxin response factor 18* (*ARF18*), and *cytochrome p450 78a9* (*CYP78A9*), respectively. Both of them have been reported to regulate cell growth to affect seed weight and silique length in rapeseed by being involved in auxin metabolism [49,50]. Little is known about *BnaA09G0559800ZS*, another gene that differs in expression between NILs. In addition to expressing differences, *ARF18* also had differences in the coding sequences between parents. Among the other three genes with coding sequence differences, *BnaA09G0559200ZS* is a homolog of Arabidopsis *VPS30* (*Vacuolar Protein Sorting 30*), which is associated with *USL1*, which regulates auxin transport [51]. Common light conditions in seedlings modulate *BnaA09G0559600ZS* homologous to *At-RS31* (*arginine/serine-rich splicing factor 31*) through retrograde signals of chloroplasts [52]. Overall, functional annotations of these candidate genes provide a good foundation for further research. Transgenic experiments will be conducted to validate the target gene in the future.

## 4. Materials and Methods

### 4.1. Plant Materials

A cross was generated between the *B. napus* genotypes ZS11 and 4D122 to develop the RIL population in this study. The F_1_ hybrid was grown in September 2015 in Wuhan and then propagated in the Xining experimental fields of the Oil Crops Research Institute, Chinese Academy of Agricultural Sciences. Using the single seed descent method, 236 F_2:6_ lines were selected to form the RIL population. For mapping *RT.A09*, eight RHLs around the initially located region and their 71 sister lines were selected from the F_6_ seeds. By selfing, the F6 RHLs produced 1137 offspring consisting of the NIL population in 2020 in Wuhan.

### 4.2. SNP Genotyping

The CTAB method was used to extract the genomic DNA of the 236 RILs and two parents from young leaves. The RIL population and two parents were genotyped using a *B. napus* 50K Illumina Infinium SNP array with 28,491 SNPs developed by Greenfafa Biotech Co., Ltd. (Wuhan, China). The SNP data were initially clustered and automatically employed with the Genome Studio program (Illumina Inc., San Diego, CA, USA) by blasting against the *Darmor-bzh* genome [35]. To improve SNP array data analysis, a procedure termed bifiltering analysis was conducted [53]. SNPs with no polymorphism between the parents, both parents being homozygous, nonparental genotypes, high missing data (> 20%), and chi-squared tests for partial separation with a P value of less than 0.01 were all filtered out sequentially in this RIL population. Then, the genotyping data of all of the filtered SNPs in the RIL population and two parents were used for further analysis.

### 4.3. Construction of the Genetic Linkage Map

After SNP filtering, valid SNPs with the same genotypes for the population were considered a “bin”. Then, the linkage group was constructed using JoinMap 4.0 [54] with a logarithm of odds (LOD) threshold of 2.0, and the SNP markers were divided into the corresponding chromosomes (A01–A10; C01–C09). Recombination frequencies were converted into map distances using the Kosambi map function. The unlinked markers were removed, and adjacent marker calculations were performed. MapChart 2.2 was used to draw the QTL position on each chromosome [55]. The quality of the genetic linkage map was checked by collinearity analysis between the genetic location and physical location of the common SNP marker at each linkage group and the corresponding chromosome. The RIL population genetic map linear analysis diagram was drawn by the MATLAB software based on the markers’ linear correlation.

### 4.4. Hydroponics Experiment

Uniform and healthy rapeseed seeds were selected for nursery development and sown on medical gauze in a blue plastic basin (60 cm length × 40 cm width × 15 cm height) (Figure S3). A quarter strength (1/4) modified Hoagland solution was added to the blue plastic basin for moisture retention and the provision of nutrients for seed germination. On the 6th day, uniform seedlings were chosen and transferred to a tiny blue plastic basin (34 cm long, 26 cm wide, 12 cm high) holding 1/4th Hoagland solution. Once a week, a half-strength and then a full-strength solution were used to replenish the solution until harvesting. The method has been described in detail by a previous study [24]. In all cases, the plants were grown in a greenhouse with 60–80% relative humidity under 16/8-h day/night cycles with 24/22 °C day/night. Artificial lighting (LED light) was used in the experiment with an intensity of 180 μmol photons m^−2^·s^−1^.

### 4.5. Traits Investigation

Phenotype identification was performed for the two parents when the plant was grown at the 3 EL, 7 EL, and 11 EL stages. Twenty-four plants were grown, and three plants were sampled at each time point within three independent repetitions. 

Four independent experiments were performed with a randomized complete block design for the RIL population in a greenhouse. In each experiment, 10 uniform and healthy rapeseed seeds of each line in the RIL population were sown in the germination device as described by a previous study [24]. At six days after germination, six seedlings of each line were transferred to the tiny blue plastic basin. Each tiny blue plastic basin was composed of four lines. As a result, 60 tubs were performed for the 236 F_2:6_ lines and two parents in an experiment. When the parent ZS11 was grown at the 3 EL stage, samples (3 plants/line) were collected. 

The PRL, RFW, and SFW were manually measured after the total roots were removed from the shoot base. The WinRHIZO software (Pro 2012b, Canada) was used to scan and analyze the undamaged roots in order to determine the TRL, TSA, TRV, and TNR. The RSR was determined as the ratio of the SFW to the RFW. After being oven-dried at 65 °C, the RDW and SDW were measured. A progeny test of 71 F6 sister lines and 14 recombinants described above was performed for the fine mapping of *RT.A09*. Twenty-four plants of each line were sampled at 3 EL, and the SFW, RFW, TRL, TSA, TRV, and TNR were investigated to evaluate the phenotype of the line.

### 4.6. Phenotypic Data Analysis and QTL Mapping

The SPSS 26.0 software was used to perform the variance (SPSS, Inc., Chicago, IL, USA). For correlation analyses, the “PerformanceAnalytics” package was utilized in R (https://github.com/braverock/PerformanceAnalytics/, accessed on 15 January 2022). The broad-sense heritability was computed using the formula reported by Liu et al. [56]. Windows QTL Cartographer version 2.5 was used to detect QTLs using the composite interval mapping (CIM) approach [57]. The detected QTLs were further integrated into clusters if the QTLs were located at the same position or the overlap interval was greater than half of the "2LOD" confidence interval and had the same additive effect (+, −). The QTL clusters, including QTLs for a trait detected in two or more environments, were considered stable QTL clusters. The naming method for each detected QTL or QTL cluster was “q” + “abbreviation of the specific trait(s)” or “qc” + “specific linkage group,” respectively. The pictures of QTL clusters on the linkage were displayed using the software MapChart 2.30.

### 4.7. Fine Mapping through PARMS Markers

To verify and narrow down the target location of the QTL *RT.A09*, seven new molecular markers based on insertion/deletion and SNP variations between two parents and four SNP markers from linkage mapping were designed using a Penta-primer amplification refractory mutation system (PARMS) by Gentides Biotech Co., Ltd. (Wuhan, China) (Table S6). These markers were first used for genotyping the 236 RIL lines and then integrated into the A09 linkage of the RIL population using JoinMap 4.0. Four SNP markers were used for genotyping all investigated progeny plants of the 71 F6 sister lines. The recombinant lines were screened using all of the markers to minimize the mapping region.

### 4.8. Candidate Gene Prediction and Real-Time PCR Analysis

Annotations of the candidate genes in the located region were obtained from the ZS11 reference genome (http://cbi.hzau.edu.cn/bnapus/, accessed on 18 October 2021). The expression patterns were first checked using the *Brassica napus* transcriptome information resource (http://yanglab.hzau.edu.cn/BnTIR, accessed on 18 October 2021). Candidate genes with expression in roots and leaves were selected for further evaluation. The expression differences of these candidate genes were evaluated using RNA extracted from roots and leaves at the 3 EL stage from NILs by real-time quantitative PCR (qRT–PCR). Three biological replications were used for each examined gene. The primer sequences used for qRT–PCR are presented in Table S8. The SYBR qPCR Master Mix (Vazyme, Nanjing, China) was used for qRT–PCR analysis with the CFX96 (BIO-RAD, Hercules, California, USA). Three technical replications were performed for each sample. *B. napus ACTIN2* was used as an internal control to compute the relative expression of target genes by the 2^−ΔΔCT^ method [58]. The CDS variations of these candidate genes were analyzed based on comparing sequencing between two parents.

## 5. Conclusions

Rapeseed is not only one of the most important vegetable oil sources for human consumption but is also the world’s second-largest source of biodiesel. Understanding the genetic architecture and molecular mechanism that affect root development is essential for determining the root system architecture, nutrient efficiency, and yield potential in rapeseed. In the present study, eight stable QTL clusters, including three major loci associated with root-related and shoot biomass traits, were identified using a newly constructed recombinant inbred line (RIL) population and a high-density linkage map in rapeseed (*B. napus* L.). One of the major QTL clusters, *RT.A09*, was fine mapped to a 129-kb interval with 19 annotated genes in the reference genome by the map-based gene cloning method. In addition, we developed codominant markers that were tightly linked with the three major QTL clusters that could be employed to improve the root system using MAS in rapeseed. Furthermore, by integrating the results of real-time PCR and comparative sequencing, our study provided five candidate genes with expression differences and/or amino acid differences for *RT.A09*. Our findings presented here could form the basis to reveal the molecular mechanism of rapeseed root development and are helpful for MAS in rapeseed breeding with excellent root systems.

## Figures and Tables

**Table 1:** Descriptive statistics for the investigated root and shoot biomass traits.

Traits	Exp	4D122	ZS11	The RIL Population							
		Mean	Mean	Min	Max	Mean	SD	CV (%)	Skw	Kur	*h^2^*
SFW (g)	1	3.652	3.702	1.731	4.805	3.054	0.513	16.8	0.42	0.49	0.83
	2	3.253	3.715 *	2.019	5.134	3.412	0.536	15.7	0.48	0.35	
	3	0.673	1.361 **	0.700	2.329	1.487	0.299	20.1	−0.01	0.31	
	4	1.616	2.477 **	1.656	4.927	3.206	0.657	20.5	−0.15	0.16	
SDW (g)	1	0.245	0.266	0.108	0.368	0.215	0.039	18.2	0.57	1.07	-
	2	0.206	0.205	0.103	0.337	0.208	0.039	18.8	0.39	0.17	
	3	0.041	0.089 **	0.039	0.150	0.082	0.019	22.6	0.10	1.18	
	4	0.084	0.175 **	0.078	0.313	0.179	0.042	23.6	−0.06	0.30	
RFW (g)	1	0.590	0.730 *	0.190	0.774	0.460	0.101	22.0	0.38	0.22	0.78
	2	0.586	0.855 *	0.301	0.814	0.545	0.100	18.3	0.43	0.01	
	3	0.098	0.290 **	0.121	0.503	0.296	0.072	24.3	0.06	0.15	
	4	0.182	0.447 **	0.210	0.838	0.474	0.110	23.2	−0.12	0.19	
RDW (mg)	1	26.3	36.0 *	9.0	36.0	21.2	4.7	22.2	0.54	0.37	-
	2	30.3	38.3 *	14.0	38.7	25.7	5.0	19.5	0.46	0.00	
	3	4.7	13.7 **	4.7	18.7	11.1	3.0	26.8	0.05	−0.26	
	4	10.0	23.3 **	8.0	33.0	18.0	4.7	26.2	0.31	0.57	
PRL (cm)	1	34.4	28.5	14.7	31.4	21.6	3.4	15.6	0.46	0.42	0.70
	2	37.6	22.2 **	14.1	37.6	22.4	4.3	19.1	0.53	0.32	
	3	26.2	24.2	14.1	40.7	28.2	4.5	15.9	0.01	0.12	
	4	29.8	27.9	17.5	40.7	28.3	4.4	15.7	0.11	0.14	
TRL (cm)	1	918.6	1244.7 *	393.1	1180.4	783.5	157.2	20.1	0.36	−0.07	0.80
	2	907.1	1042.3	427.0	1308.2	714.8	139.7	19.5	0.74	1.16	
	3	211.2	448.0 **	246.1	836.7	534.7	111.6	20.9	−0.21	0.02	
	4	526.5	834.7 **	449.0	1418.4	859.5	179.5	20.9	0.12	0.34	
TSA (cm^2^)	1	69.0	85.3	28.3	80.0	52.8	9.76	18.5	0.43	0.15	0.79
	2	72.8	94.4 *	41.2	106.8	62.9	10.38	16.5	0.87	1.50	
	3	13.2	29.5 **	16.7	53.1	34.7	7.25	20.9	−0.18	0.07	
	4	31.0	55.0 **	28.0	84.9	56.2	11.34	20.2	−0.19	0.29	
TRV (cm^3^)	1	0.413	0.466	0.144	0.479	0.286	0.059	20.8	0.58	0.33	0.78
	2	0.465	0.681 *	0.257	0.693	0.445	0.075	16.9	0.58	0.44	
	3	0.066	0.155 **	0.088	0.290	0.180	0.040	22.2	−0.04	0.07	
	4	0.145	0.289 **	0.136	0.440	0.293	0.063	21.5	−0.21	0.22	
TNR	1	1042	1367	461	1812	976	253.2	25.9	0.42	0.11	0.61
	2	842	721	380	1461	648	157.6	24.3	1.01	2.56	
	3	572	741 *	302	2716	1121	400.6	35.7	0.90	0.97	
	4	1239	1317	581	2568	1189	344.3	29.0	0.82	0.99	
RSR	1	0.162	0.197 *	0.075	0.264	0.152	0.030	19.5	0.60	0.50	0.80
	2	0.191	0.226 *	0.108	0.240	0.161	0.025	15.5	0.36	−0.09	
	3	0.145	0.215 **	0.120	0.331	0.199	0.032	15.8	0.33	0.78	
	4	0.113	0.181 **	0.094	0.235	0.149	0.024	16.3	0.32	0.20	

Exp, Experiment; Min, Minimum in the population; Max, maximum; Mean, mean trait value; SD, Standard deviation of trait values; CV, coefficient of variation; Skw, Skewness; Kur, Kurtosis; *h*^2^, Heritability. ** and * indicated significant differences of the trait between two parents at *p* < 0.01 and *p* < 0.05, respectively.

**Table 2:** Summary of stable QTL clusters detected for root-related and shoot biomass traits in the RIL population across four independent experiments.

Stable QTL Cluster	Chr	Traits	Exp	Peak Position	Confidence Interval	Physical Position (Mb)	Add	Max LOD	Max R^2^ (%)
qcA01-2	A01	PRL	1, 3	30.21	22.3–40.5	2.80–6.15	−	4.7	7.1
qcA09-1	A09	SDW	1, 3	8.81	5.8–10.8	0.59–1.79	−	4.2	6.5
qcA09-2	A09	SFW, SDW, RFW, RDW, TSA, TRV	1, 3, 4	105.41	88.0–115.0	24.57–28.77	−	6.9	10.8
qcC02-1	C02	SDW	1, 2	13.41	10.1–16.8	11.89–16.21	+	4.7	6.7
qcC02-2	C02	PRL	1, 2	21.41	17.2–33.3	17.07–35.86	−	3.7	5.4
qcC03-1	C03	SFW, SDW	1, 2, 3	108.81	86.9–122.1	23.35–49.99	−	6.0	9.1
qcC08-2	C08	SFW, SDW, RFW, TRL, TSA, TRV	2, 3, 4	40.41	29.9–44.8	20.08–25.30	+	9.0	14.1
qcC08-3	C08	PRL, TRL, RSR	1, 2, 4	95.01	72.9–99.3	34.07–37.35	+	9.0	13.9

Chr, Chromosome; Exp, Experiment. Add, “+”, and “−” represent corresponding QTLs that carried positive additive effects from the 4D122 and ZS11 alleles, respectively.

**Table 3:** Information on the candidate genes in the target region of *RT.A09*.

Gene ID in ZS11	Gene Position in ZS11	Homologs in Arabidopsis	Function Annotation	Expression in Root and Leaves	CDS Differences	aa Differences
BnaA09G0558900ZS	57306631–57309156	AT3G61720.1	MCTP12, Ca^2+^ dependent plant phosphoribosyltransferase family protein	No	-	-
BnaA09G0559000ZS	57311257–57312591	AT2G25410.1	ATL22, RING/U-box superfamily protein	No	-	-
BnaA09G0559100ZS	57317117–57318844	AT3G61750.1	Cytochrome b561/ferric reductase transmembrane with DOMON related domain	Yes	10 SNP	No
BnaA09G0559200ZS	57320270–57321154	AT3G61770.1	VPS30, Acid phosphatase/vanadium-dependent haloperoxidase-related protein	Yes	9 bp insertion, 7 SNP	3 aa insertion, 3 aa substitute
BnaA09G0559300ZS	57331807–57334821	AT3G61830.1	ARF18, auxin response factor 18	Yes	6 bp insertion, 22 SNP	2 aa insertion, 20 aa substitute
BnaA09G0559400ZS	57335044–57335702	AT3G61840.1	auxin response factor, Protein of unknown function (DUF688)	Negligible	-	-
BnaA09G0559500ZS	57338272–57340276	AT3G61850.4	Dof-type zinc finger DNA-binding family protein	Yes	1 SNP	No
BnaA09G0559600ZS	57344816–57345545	AT3G61860.1	RSP31, an arginine/serine-rich splicing factor	Yes	11 bp deletion, 5 SNP	translation frameshift
BnaA09G0559700ZS	57346730–57347450	AT5G17370.2	Transducin/WD40 repeat-like superfamily protein	No	-	-
BnaA09G0559800ZS	57348205–57349656	AT2G46620.1	P-loop containing nucleoside triphosphate hydrolases superfamily protein	Yes	7 SNP	No
BnaA09G0559900ZS	57353926–57355199	AT3G61870.1	-	Yes	No	No
BnaA09G0560000ZS	57355937–57356626	AT2G46630.1	-	Yes	No	No
BnaA09G0560100ZS	57359705–57361780	AT3G61880.2	CYP78A9, cytochrome p450 78a9	Yes	No	No
BnaA09G0560200ZS	57396791–57397098	AT5G63200.1	tetratricopeptide repeat (TPR)-containing protein	No	No	No
BnaA09G0560300ZS	57401071–57401430	AT3G61900.1	SAUR33, SAUR-like auxin-responsive protein family	Yes	No	No
BnaA09G0560400ZS	57418544–57419110	AT3G61920.1	-	Yes	1 SNP	No
BnaA09G0560500ZS	57421745–57422077	AT3G61930.1	-	No	-	-
BnaA09G0560600ZS	57424170–57425177	AT3G61940.1	MTPA1, Member of Zinc transporter (ZAT) family	No	-	-
BnaA09G0560700ZS	57426789–57427774	AT3G61950.1	MYC67, MYC-type transcription factor	No	-	-

Gene ID: Gene models annotated in the ZS11 reference genome (http://cbi.hzau.edu.cn/bnapus/, accessed on 18 October 2021); Expression in root and leaves: expression patterns of these genes in roots, vegetative rosette organs, stem, and leaves checked using the BnTIR database (http://yanglab.hzau.edu.cn/BnTIR/, accessed on 18 October 2021) (Table S7); CDS differences: differences in coding sequence between two parents; aa differences: differences of amino acids between two parents.

## Data Availability

The datasets generated during and/or analyzed during the current study are available from the corresponding author on reasonable request.

## Supplementary Materials

The following supporting information can be downloaded at: https://www.mdpi.com/article/10.3390/ijms23094781/s1.
