# Supplementary material for: Discovery of Genomic Regions and Candidate Genes Controlling Root Development Using a Recombinant Inbred Line Population in Rapeseed (Brassica napus L.)
Source: Int J Mol Sci. 2022 Apr 26;23(9):4781. doi: 10.3390/ijms23094781 (PMC9102059; doi:10.3390/ijms23094781)
Supplement: Supplementary file 1 [file ijms-23-04781-s001.zip › List of Figs(S).pdf]

Figure S1

RHL	chrA09_27836192 (A9-1)	chrA09_27935312 (A9-2)	chrA09_28188769 (A9-3)	chrA09_28221466 (A9-4)	No. of F_6 lines
R4	H	H	H	H	7
R19	H	H	H	H	10
R26	H	H	H	H	9
R41	A	H	H	H	9
R48	H	H	B	B	9
R78	H	H	H	H	10
R159	H	H	H	H	9
R183	H	H	H	H	8

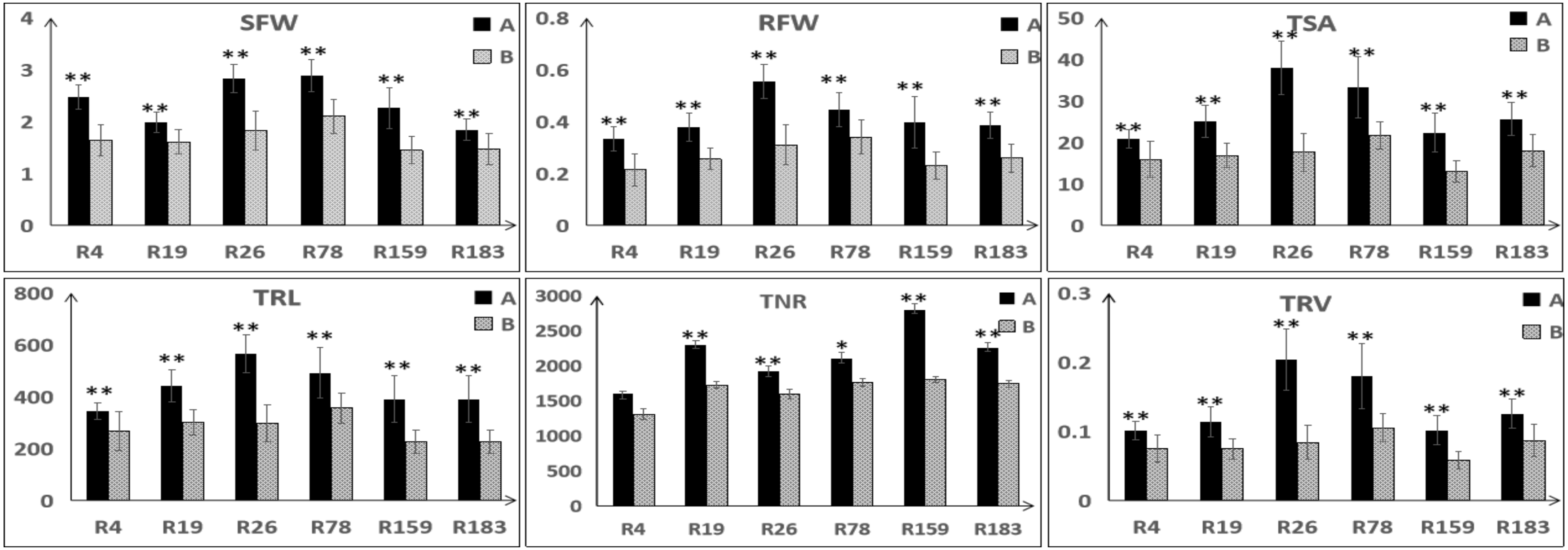

The differences of the SFW, RFW, TRL, TSA, TNR, and TRV between NIL-A and NIL-B for six residual heterozygous lines (RHLs) by progeny test. “A” and “B” indicate that the genotype of this line on this marker corresponded to ZS11 and 4D122, respectively. “H” indicates that the genotype of this line on this marker was heterozygous. ** and * indicate significant differences at P < 0.01 and P < 0.05, respectively.

Figure S2

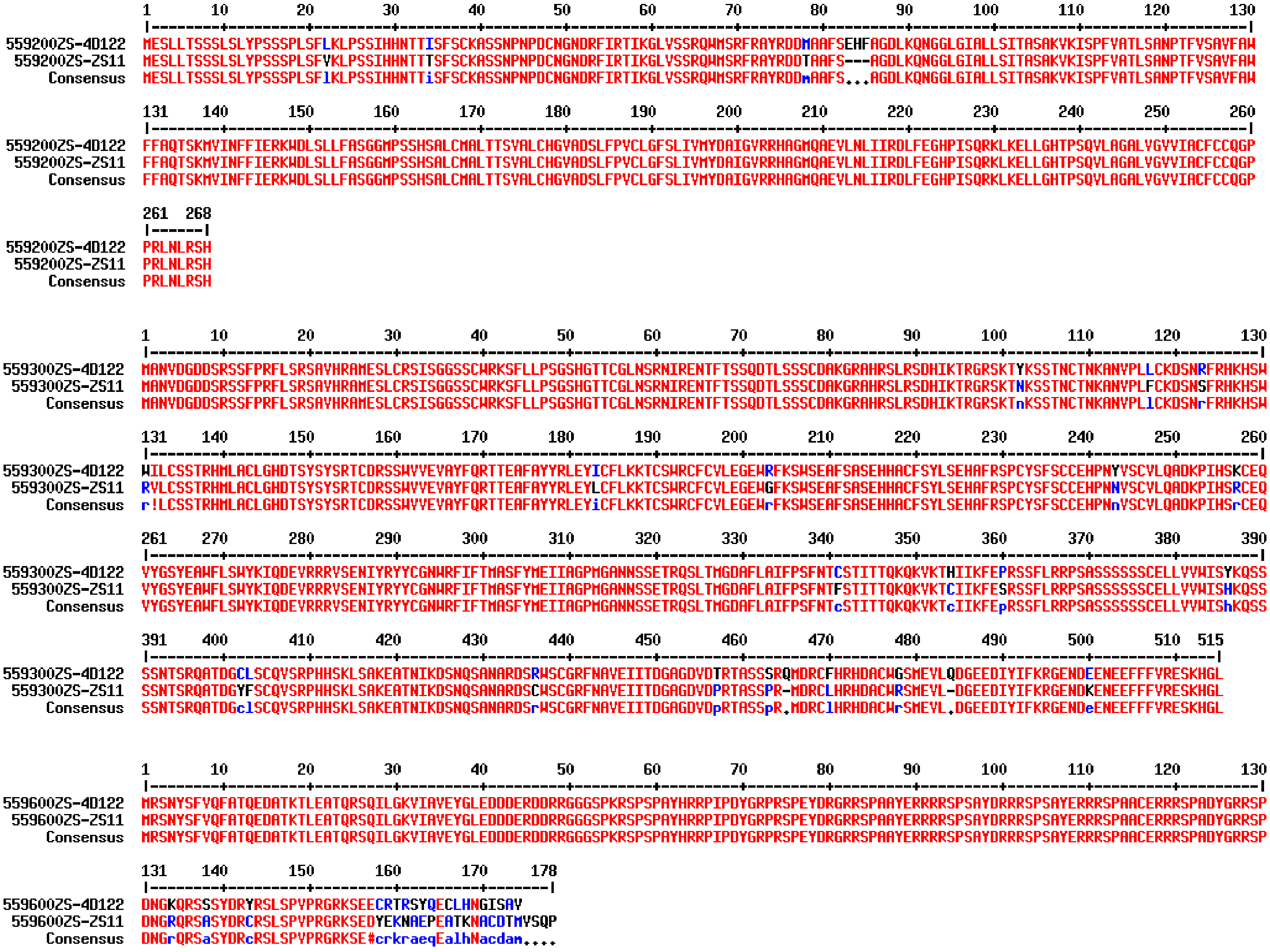

The sequence comparisons of amino acids for the three candidate genes.

Figure S3

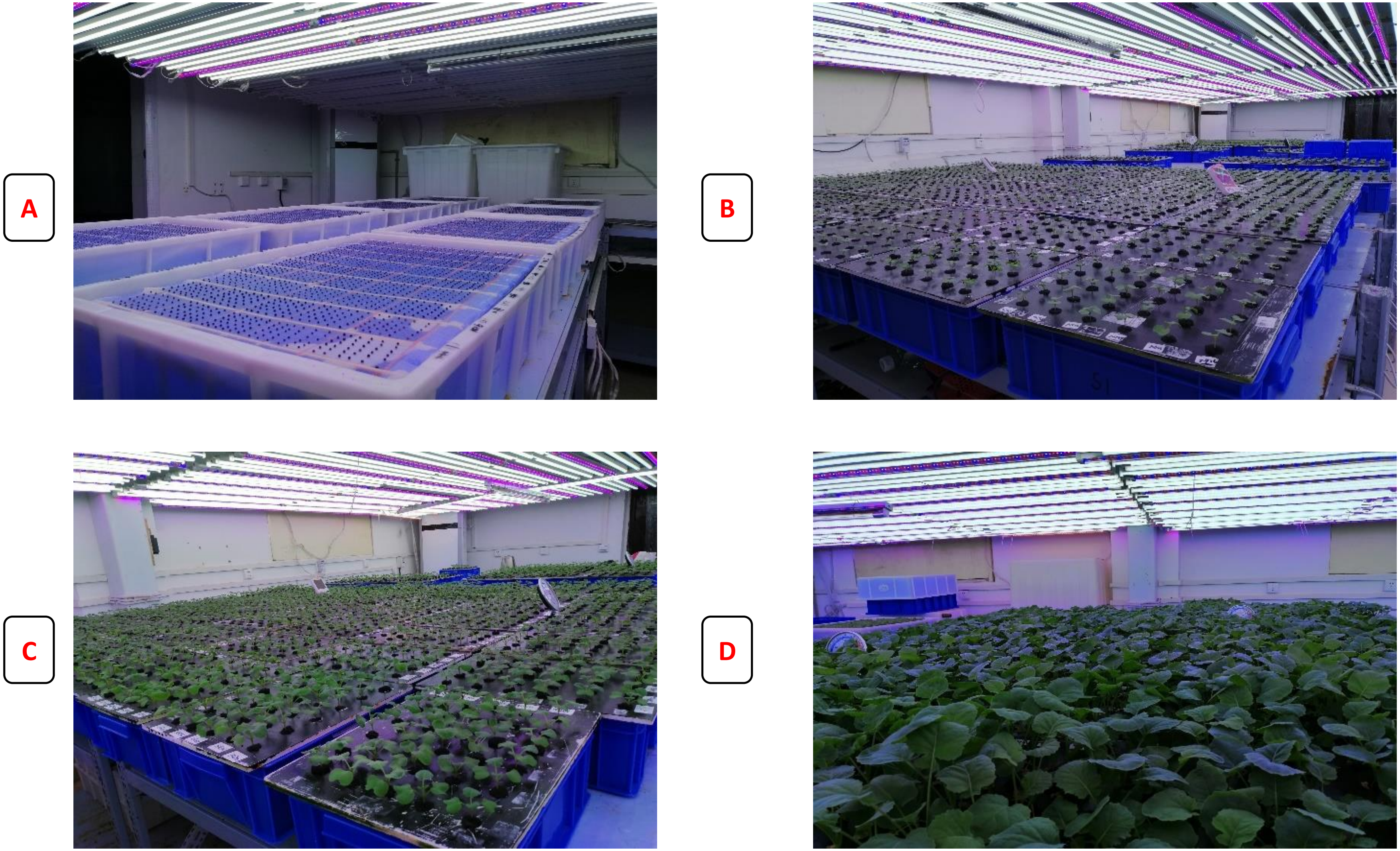

Growing of plants at different stages (A) Materials sowed on germination device (germination stage). (B) Plants in the growth device six days after transplantation (transplanting stage). (C) Plants in the growth device at 1/2 changing solution (tiny blue plastic basin). (D) Plants at sampling stage (18 days old seedlings)
